# A Rapid, Cost-Effective Method of Assembly and Purification of Synthetic DNA Probes >100 bp

**DOI:** 10.1371/journal.pone.0034373

**Published:** 2012-04-06

**Authors:** Michael A. Jensen, Lauren Jauregui, Ronald W. Davis

**Affiliations:** Stanford Genome Technology Center, Palo Alto, California, United States of America; Université Paris-Diderot, France

## Abstract

Here we introduce a rapid, cost-effective method of generating molecular DNA probes in just under 15 minutes without the need for expensive, time-consuming gel-extraction steps. As an example, we enzymatically concatenated six variable strands (50 bp) with a common strand sequence (51 bp) in a single pool using Fast-Link DNA ligase to produce 101 bp targets (10 min). Unincorporated species were then filtered out by passing the crude reaction through a size-exclusion column (<5 min). We then compared full-length product yield of crude and purified samples using HPLC analysis; the results of which clearly show our method yields three-quarters that of the crude sample (50% higher than by gel-extraction). And while we substantially reduced the amount of unligated product with our filtration process, higher purity and yield, with an increase in number of stands per reaction (>12) could be achieved with further optimization. Moreover, for large-scale assays, we envision this method to be fully automated with the use of robotics such as the Biomek FX; here, potentially thousands of samples could be pooled, ligated and purified in either a 96, 384 or 1536-well platform in just minutes.

## Introduction

Synthetic DNA probes are widely used in many assays today including massively parallel SNP detection, cancer genome resequencing, proximity ligation, and gene knockout studies [1]–[3]. Typically probes are between 100 and 120 bp, the length of which may constitute regions of homology with the target, forward and reverse primer sequences for amplification, and a barcode for identification (e.g. molecular inversion probe design [4]). Within the last decade, advances in chemistry and instrumentation development of oligodeoxynucleotide (ODN) synthesis have made it possible to generate strands >100 bp with coupling efficiencies (CE) 99% or higher. However, synthesis of whole strands this size has its drawbacks which include: 1) the longer the probe, the more failure sequences result (full-length product (FLP) generation = (CE^n-1^)). For example, synthesis of a 101 mer at a coupling efficiency of 99% yields ∼37% FLP; the remaining ∼64% must be removed by means of purification to prevent n-1 species from contaminating the downstream application, and 2) synthesis time for probes >100 bp can easily exceed 10 hr, and often technicians are not available to monitor the entire run, and therefore cannot catch an instrumentation failure or replenish consumables before they are exhausted. The latter can cost the end-user hundreds of dollars in time and reagents with the additional costs of having to repeat the synthesis. On the other hand, the notion of ligating shorter constituent fragments into a full-length, single-stranded probe, has its advantages such as 1) synthesis time and reagent consumption are both cut in half, 2) a crude 50 mer with a 99% CE, yields ∼61% FLP compared to only 37% for a 101 mer, and 3) in cases where probes share a common sequence (e.g. universal primer regions for PathogenMIP detection [5], reagent consumption is further reduced by requiring only the variable regions, one universal strand and one complimentary bridge for assembly.

The current method of ligating shorter strands into full-length probes [5]–[7] follows the general scheme of concatenating two fragments of approximately equal length using a thermal-stable ligase in combination with a bridge; the FLP is then separated out from the unincorporated species by gel-extraction using Agarose, Capillary [8] or polyacrylamide gel electrophoresis (PAGE). While the ligation reaction can take anywhere from 20 to 60 min with cycling parameters, the process of isolating FLP can take up to 4 hr (including gel preparation, sample loading, electrophoresis, and sample extraction/purification).

Here we introduce a rapid, cost-effective alternative method of generating single-stranded ODN>100 bp in under 15 min. After room temperature (RT) ligation, the reaction mixture is then filtered through a size-exclusion column to remove all unincorporated species without the use of laborious, expensive, and low yielding extraction methods. As such, the end-user can acquire empty, recycled desalting columns or barrier-style pipette tips to load with the polymeric resin for sample purification. Furthermore, we have eliminated the need for a thermal cycler, gel electrophoresis apparatus, Agarose/PAGE pre-casts, voltage box and kit for gel extraction, all of which could save the end-user thousands of dollars in sample preparation.

## Materials and Methods

### ODN Synthesis

ODN synthesis was done in-house (Stanford Genome Technology Center) with an ABI 3900 DNA/RNA synthesizer (Applied Biosystems) using 1000 Å CPG columns (Biosearch Technologies) for a 50 nm-scale synthesis. Cycle conditions were similar to manufacture's recommended protocol, which included the following reagents: deblock (3% TCA/DCM) (AiC), acetonitrile, 0.02 M oxidizing solution, cap A/B, 0.1 M solutions of dA, dC, dG and dT (Proligo), and 0.25 M 5-Benzylthio-1H-tetrazole (Glen Research). Post-synthesis steps included ODN cleavage from the support followed by base-deprotection overnight at 55°C with ammonium hydroxide (28–30%) (J.T. Baker). After lyophilization, ODNs were resuspended and the optical density for each was measured via Spectramax 384 Plus 96-well plate reader at 260 λ.

### ODN Analysis

All ODNs were normalized to 1000 µM in water, and analyzed for purity using reverse-phase high-performance liquid chromatography (HPLC) with UV detection at 260 λ; running buffers consisted of water, acetonitrile, triethyl ammonium acetate and EDTA (HPLC unit and operating software used for sample analysis consisted of the Transgenomic Wave System).

### Probe design

Each 101 bp probe consists of a common strand (51 bp), variable (50 bp), and a bridge (20 bp); the bridge in turn, shares homology with 10 bases of the variable 3′ end and 10 bases at the 5′ end of the common strand. In addition to the sequences listed in Table 1 (S1, S2, S3, S4, S5, S6), we tested several other combinations to further validate the process (data not shown).

**Table 1:** Sequences used for generating 101 bp probes (variables, S1–S6, were pooled with the complimentary bridge and common strand in the presence of Fast-Link/Quick Ligase).

Sample	Sequence (5′ – 3′)
Bridge_20 bp	***CTGAACCGCTCTTCCGATCT***
Common strand_51 bp	***AGCGGTTCAG***CAGGAATGCCGAGACCGATCTCGTATGCCGTCTTCTGCTTG
Random Variable 50 bp S1	TGCAAGTCCAGTTTTACTGGAACACACAGCACGCTCCCTT***AGATCGGAAG***
Random Variable 50 bp S2	CAACATTAGCCTTAGTTCCCTATTGATTGTGATGCCATCA***AGATCGGAAG***
Random Variable 50 bp S3	CAAGACAAGTTCACAGAGTTAGAATGAGTAGTTTAATATT***AGATCGGAAG***
Random Variable 50 bp S4	ATGTAGTTTGGGTCCAGGAAGAAACAAGGCTTGGGGTCCA***AGATCGGAAG***
Random Variable 50 bp S5	AATAATACAGCTGGGGACGACCTGGCCAAGCTGCTGTGGC***AGATCGGAAG***
Random Variable 50 bp S6	GTACTGGAAGTTTCAAGGTTTTGGAAAACAAGCAATTCTC***AGATCGGAAG***

### Ligation reaction

Prior to ligation, common strands were enzymatically 5′ phosphorylated by adding 3 ul (∼3000 pm) DNA to 41 µl water, 5 µl 10× T4 DNA ligase buffer with ATP (NEB) and 10 U T4 Polynucleotide Kinase (NEB) for a 50 µl final volume. The mixture was incubated at 37°C for 30 min then heat-inactivated at 60°C for 20 min using the Veriti 96-well thermal cycler (Applied Biosystems).

Six µl variable (1 µl each), 6 µl bridge and 6 µl common strand were added in equimoloar amounts (1000 pm/µl) to 22 µl water, 5 µl buffer, 3 µl ATP, 2 µl Fast-Link™ DNA ligase (Epicentre) for a total reaction volume of 50 µl. Reactions were carried out for 10 min at RT; FLP was then purified from the unligated species by both gel-extraction and size-exclusion methods to compare their relative yield and purity.

### Sample purification

For separating FLP from the unligated product, we used Bio-Gel P-10 Polymeric resin (BIO RAD) with a size-exclusion limit of 20,000 daltons. Eight-hundred µl of slurry (1 gm P-10/10 ml in water) were dispensed into empty BIO RAD desalting columns (P-6 or P-30). Approximately 200 µl of water were removed from the column by applying a slight vacuum—enough to uniformly package the resin to prevent air pockets. Afterward, columns were placed inside 2 ml collection tubes and centrifuged at 2400 RPM for 2 min; the resultant water was then discarded. The complete reaction mixture (50 µl) was then added to the resin bed and then centrifuged again for 2 min (2000 RPM). In addition, we chose two popular gel-extraction methods to compare both yield and purity with that of our size-exclusion process.

## Results and Discussion

**Figure 1:**
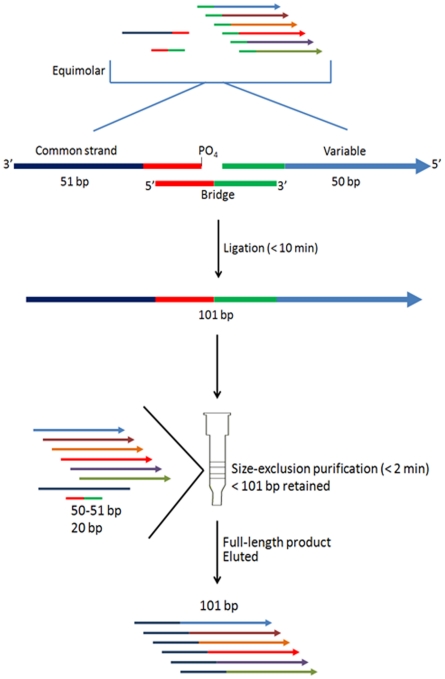
Figure shows the general process of probe ligation followed by FLP size-exclusion purification. To make each probe, the variable, bridge and common strands are first combined in the presence of a ligase mix (e.g. Fast-Link or Quick Ligase); the reaction is then carried out at RT for 10 min. Afterward, the sample is passed through P-10 polymeric resin where unincorporated species are thus retained, and FLP eluted. The final purified, desalted product of pooled ODN probes is now ready for downstream application.

Molecular probes are essential to many biological applications including interrogation of tumorigenic polymorphisms, as well as for identifying pathogens of interest. For such large-scale, multiplexed assays where thousands of probes are used, cost can become a limiting factor. Typically, strands in the range of 100—120 bp are made chemically via DNA synthesis automation; however, the longer the strand synthesized, the more failure sequences are produced. Consequently, samples often require PAGE purification to isolate the FLP, which can be very costly. One avenue taken to circumvent this drawback is by enzymatically ligating two shorter constituents of ∼equal length to generate the full-length probe. Current methods are generally time-consumptive (∼5 hr) and require expensive laboratory equipment and extraction kits to purify the end-product. As such, the focus of our paper has been to detail an alternative method of assembling and purifying ODNs>100 bp, a process that takes just under 15 min (outline given in Figure 1).

**Figure 2:**
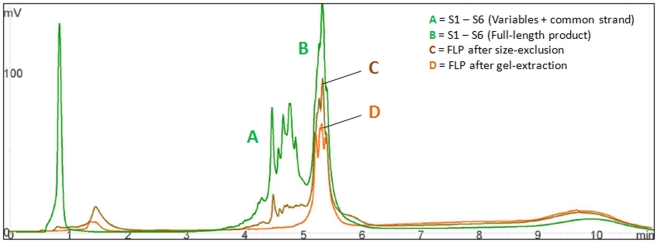
Chromatograms comparing purity and yield of final product before and after purification. A = unincorporated common and variable strands, B = FLP, C = S1–S6 after size-exclusion purification, and D = S1–S6 after gel extraction.

Figure 2 shows a chromatogram of a crude ligation reaction with A) unincorporated variable and common strands and B) 101 bp FLP (S1–S6) between 5 and 5.5 min. Superimposed on to this image is C) the purified product after filtration through a size-exclusion P-10 polymeric resin, with a final yield three-quarters that of the initial reaction compared to D) standard gel-extraction method, with a final yield at about one-half. Though there are some residual common and variable strands following the size-exclusion purification, it is significantly reduced.

We tested several ligation pools of random variables with different common strands and bridges, and found the overall yields between crude and purified samples comparable to those shown in Figure 2. However, yield for samples after gel-extraction varied considerably where FLP return was never greater than 50%, and often as low as 25% of the initial full-length amount.

For this work, the process of phosphorylating the common strands was done enzymatically, which required the use of a thermal cycler; however, whether the strands are purchased from an outside vendor or made in-house, the 5′ phosphate group can be coupled directly onto the ODN using a phosphorylation reagent (cheapest of all 5′ ODN modifiers).

While both Fast-Link and Quick ligase mixes were examined for this research, and shown to perform equally well, Quick ligase does have one drawback. When separating out FLP from the unincorporated species through a size-exclusion column, the polyethylene glycol (PEG) in the buffer had a leaching effect on the resin; when hydrated, the sample was opaque, and when lyophilized, a significant amount of precipitation occurred where the remaining pellet could not be dissolved. Alternatively, we did not observe any precipitation with Fast-Link, the buffer of which was absent of PEG. Therefore, this made the latter an obvious choice for our ligation method using size-exclusion purification.

As for modifying the ligation reactions to maximize incorporation of common and variable strands into FLP, we found no benefits of 1) increasing ligation times above 10 min, 2) varying reaction temperatures 3) cycling parameters, 4) annealing samples prior to ligation (e.g. boil, then slowly cool to RT over 15 min), or 5) varying bridge lengths above or below 20 bp; consequently, the one determining factor of ligation efficiency has been to achieve a 1∶1∶1 ratio of reactants. And while initial normalization of reactants is essential, it is the most challenging step of the assay; no matter how careful we were in measuring sample concentrations, there was usually some residual variable, bridge and common strand leftover as evidenced by HPLC. This discrepancy can likely be a product of an improperly calibrated pipettor or manual handling error, both of which may be resolved by sample automation. Nevertheless, this has necessitated our use of a method for purifying the FLP from the unligated species as to prevent any possible contamination during downstream application.

Though we determined operational centrifugation time and speed for purifying 101 mers using the P-10 resin, the overall procedure could benefit from further optimization. This includes adjusting 1) resin loading (column resin-bed height and more uniform packaging), 2) RPM, 3) spin time, and 4) resuspending the P-10 slurry in a storage buffer that is [salt] and pH specific. Moreover, cost and time may be further decreased by scaling-up the ligation reactions from 6 variables per pool to 12 or more. The only caution we have is during the purification process; overloading the column with sample could have a negative effect on final yield. To compensate for this, we suggest lowering the final reactant concentrations accordingly.

### Conclusion

Here we introduce a quick and cost-effective method for assembling and purifying synthetic DNA probes >100 bp. As an alternative to currently used protocols, our ligation reactions are carried out at RT for only 10 min, and the process of purifying FLP from unligated species is done simply by passing the reaction mixture through a size-exclusion column, which takes less than five minutes. As such, we eliminate the need for costly laboratory equipment including a thermal cycler and gel electrophoresis apparatus; expensive sample extraction kits are also unnecessary. Furthermore, the end-user only requires recycled desalting columns or barrier-style pipette tips for loading with P-10 resin for purifying the ligation reaction. Moreover, for large-scale assays, we envision this method to be fully automated with the use of robotics such as the Biomek FX; here potentially thousands of samples could be pooled, ligated and purified in either a 96, 384 or 1536-well platform in just minutes.
